# Dengue Virus Capsid Protein Usurps Lipid Droplets for Viral Particle Formation

**DOI:** 10.1371/journal.ppat.1000632

**Published:** 2009-10-23

**Authors:** Marcelo M. Samsa, Juan A. Mondotte, Nestor G. Iglesias, Iranaia Assunção-Miranda, Giselle Barbosa-Lima, Andrea T. Da Poian, Patricia T. Bozza, Andrea V. Gamarnik

**Affiliations:** 1 Fundación Instituto Leloir-CONICET, Buenos Aires, Argentina; 2 Instituto de Bioquímica Médica, Universidade Federal do Rio de Janeiro, Rio de Janeiro, Brazil; 3 Laboratório de Imunofarmacologia, Fundação Oswaldo Cruz, Rio de Janeiro, Brazil; Washington University School of Medicine, United States of America

## Abstract

Dengue virus is responsible for the highest rates of disease and mortality among the members of the *Flavivirus* genus. Dengue epidemics are still occurring around the world, indicating an urgent need of prophylactic vaccines and antivirals. In recent years, a great deal has been learned about the mechanisms of dengue virus genome amplification. However, little is known about the process by which the capsid protein recruits the viral genome during encapsidation. Here, we found that the mature capsid protein in the cytoplasm of dengue virus infected cells accumulates on the surface of ER-derived organelles named lipid droplets. Mutagenesis analysis using infectious dengue virus clones has identified specific hydrophobic amino acids, located in the center of the capsid protein, as key elements for lipid droplet association. Substitutions of amino acid L50 or L54 in the capsid protein disrupted lipid droplet targeting and impaired viral particle formation. We also report that dengue virus infection increases the number of lipid droplets per cell, suggesting a link between lipid droplet metabolism and viral replication. In this regard, we found that pharmacological manipulation of the amount of lipid droplets in the cell can be a means to control dengue virus replication. In addition, we developed a novel genetic system to dissociate cis-acting RNA replication elements from the capsid coding sequence. Using this system, we found that mislocalization of a mutated capsid protein decreased viral RNA amplification. We propose that lipid droplets play multiple roles during the viral life cycle; they could sequester the viral capsid protein early during infection and provide a scaffold for genome encapsidation.

## Author Summary

Dengue virus is the single most significant arthropod-borne virus pathogen in humans. In spite of the urgent medical need to control dengue infections, vaccines are still unavailable, and many aspects of dengue virus biology and pathogenesis remain elusive. We discovered a link between dengue virus replication and ER-derived organelles known as lipid droplets (LDs). Dengue infection increases the amount of LDs per cell and pharmacological inhibition of LD formation greatly reduces dengue virus replication. In addition, we have found that the viral capsid protein in infected cells accumulates on the surface of LDs. Manipulation of infectious clones and generation of new reporter dengue viruses allowed us to define the molecular basis of capsid protein association to LDs. Specific amino acids on the α2 helix, located in the center of the capsid protein, were found to be crucial for both accumulation of capsid protein on LDs and dengue virus infectious particle formation. We propose that LDs facilitate viral replication providing a platform for nucleocapsid formation during encapsidation. Our findings begin to unravel the complex mechanism by which dengue virus usurps cellular organelles to coordinate different steps of the viral life cycle.

## Introduction

The genus *Flavivirus* comprises a large group of emerging and re-emerging pathogens capable of causing severe human diseases. It includes yellow fever (YFV), dengue (DENV), West Nile (WNV), tick borne encephalitis (TBEV), and Japanese encephalitis (JEV) viruses. DENV is the most significant mosquito borne human viral pathogen worldwide. It infects more than 50 million people each year, resulting in around 25,000 deaths. The lack of vaccines and antivirals against DENV leaves the 2 billion people at risk, mainly in poor countries, in a constant state of alarm [1].

The replication cycle of different members of the *Flavivirus* genus is fundamentally similar. The viral genome is a single plus-stranded RNA molecule that serves as messenger for viral protein synthesis, template for RNA amplification, and substrate for encapsidation [2]. In recent years, a number of cis-acting RNA elements have been identified in the coding and uncoding regions of the flavivirus genomes as promoters, enhancers, and cyclization signals necessary for efficient amplification of the viral RNA (for review see [3]). A mechanism by which the viral polymerase specifically recognizes and copies the viral genome has been recently proposed [4]. In contrast, little is known about the recognition of the viral RNA by the capsid (C) protein. For flaviviruses, it is still unclear how, when, and where the C protein recruits the viral RNA during viral particle morphogenesis. In this work, we used DENV to investigate how the C protein usurps cellular organelles to facilitate viral replication.

The flavivirus genomes contain a long ORF encoding a polyprotein that is cleaved into three structural proteins (C, prM, and E) and seven nonstructural proteins (NS1-NS2A- NS2B-NS3-NS4A-NS4B-NS5) [5]. The proteins C and prM are connected by an internal hydrophobic signal sequence that spans the ER membrane and is responsible for the translocation of prM into the ER lumen. The first cleavage is accomplished by the viral NS3/2B protease, which resides in the cytoplasmic side of the ER membrane and separates the mature C protein from its membrane anchor sequence [6]–[8]. It has been proposed that the mature form of the C protein remains associated to intracellular membranes via an internal hydrophobic region conserved in all flaviviruses [9].

In flavivirus infected cells, the C protein was detected both in the cytoplasm and the nucleus [10]–[13]. Inside the nucleus it has been shown to accumulate in the nucleolus. The cytoplasmic fraction of the C protein of kunjin virus (KUNV) was found near structures called convoluted membranes in close association with vesicle packets, which are the sites of RNA replication [11],[14],[15]. A recent report has demonstrated a complex membrane architecture that links flavivirus genome replication and viral assembly [16]. A coupling between RNA synthesis and RNA encapsidation has been also suggested [17]. It was shown that viral RNAs were not encapsidated if they were not actively synthesized in the replication complexes. Interestingly, a complex connection between the encapsidation process and proteins of the RNA replication machinery is emerging. Specific amino acids changes in NS2A and NS3 were found to impair particle formation [18]–[21]. Whether these NS proteins bind to the C protein, to the viral RNA, or to cellular components (proteins or membranes) is still unknown.

The mature C is a highly basic protein of 12 kDa that forms homodimers in solution [22],[23]. The first 32 and the last 26 residues of the KUNV C protein were proposed to interact with the viral RNA [24]. The tridimensional structures of DENV and WNV C proteins were recently solved by NMR and crystallography, respectively [25],[26]. These studies indicated that the monomer contains four alpha helices (α1 to α4). The first 20 amino acids are unstructured in solution and were cleaved in the WNV C crystals [26]. The first 3 helices (α1 to α3) form a right handed bundle that comprises the monomer core. The different orientation of α1 in WNV and DENV suggested that this helix is flexible. The α4, the longest helix, extends away from the monomer core and has a high density of basic residues on the solvent accessible surface, which were proposed to interact with the viral RNA. On the opposite side of the molecule, the surface contributed by α2−α2′ and α1−α1′ is largely uncharged and is proposed to interact with membranes [25]. The originally described internal hydrophobic region, residues 46 to 66 in DENV C, includes helices α2 and α3 [9]. Although the C protein is the least conserved of the flavivirus proteins, the structural properties are very similar and the charge distribution is well conserved.

Here, we investigated the subcellular localization of the C protein in DENV infected cells and found that the cytoplasmic C accumulates around ER-derived organelles called lipid droplets (LDs). A novel reporter system was developed, which allowed us to dissociate cis-acting signals for RNA synthesis from the C coding sequence. Using infectious DENV RNAs and the new reporter system, specific residues in the α2 helix of the C protein were identified as crucial determinants for LD localization and DENV particle formation. Furthermore, we report that pharmacological inhibition of LD formation greatly decreases DENV replication, providing new ideas for antiviral strategies.

## Results

### Lipid droplet localization of DENV C protein in infected cells

**Figure 1:**
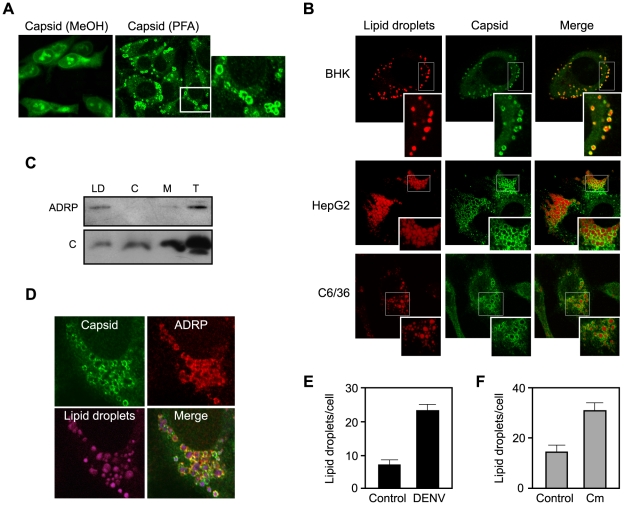
DENV infected cells accumulate the C protein around lipid droplets. A. Nuclear and cytoplasmic distribution of C protein in DENV infected BHK cells. Cells were infected with DENV2 and analyzed by immunofluorescence using a polyclonal anti-C antibody. Cells were fixed with methanol (MeOH) or paraformaldehyde (PFA) as indicated on the top. B. The C protein is targeted to lipid droplets. BHK, HepG2, and C6/36 cells were infected with DENV2, fixed at 48 h post-infection, probed with anti-C antibodies and BODIPY for lipid droplets staining, and examined by confocal microscopy. C. Subcellular fractionation of LDs. DENV-infected cell lysates were fractionated into lipid droplets (LD), cytosol (C), and microsome (M) fractions by sucrose gradient centrifugation. A total cytoplasmic extract was also included (T). The samples were immunoblotted with anti-ADRP and anti-C antibodies. D. Co-localization of C and ADRP on LDs. DENV infected BHK cells were analyzed by immunofluorescence with anti-ADRP and anti-C antibodies, and stained with BODIPY. E. DENV infection increases the number of lipid droplets. The amount of lipid droplets in control or DENV infected BHK cells were determined. Cells were fixed 48 h post- infection, incubated in 1.5% of OsO_4_, and lipid bodies were enumerated by light microscopy in 50 consecutive cells in each slide in triplicates. The bars indicate the standard error of the mean (+/−SEM), (P<0.0002). F. Expression of C protein increases the number of lipid droplets. The amount of lipid droplets in control or C expressing BHK cells were determined as described above. The bars represent the standard error of the mean (P<0.0001).

Localization of the C protein in the cytoplasm and the nucleus of DENV infected cells has been previously reported. The nuclear localization was carefully analyzed by several groups [12],[13]. In contrast, there is limited information regarding the distribution of the C protein in the cytoplasm of the infected cell, which is the place of viral encapsidation. To investigate the subcellular localization of the C protein during viral replication, DENV2 was used to infect BHK cells. As previously described, when cells were fixed with methanol and used for indirect immunofluorescence, the C protein was found in the nucleus and accumulated in the nucleolus (Fig. 1A, left panel). Methanol fixation is known to extract cellular lipids. Therefore, in order to preserve the membranous structures induced by viral infection, and to investigate the distribution of C in the cytoplasm, DENV infected cells were fixed with paraformaldehyde and permeabilized with a low concentration of Triton X-100. Remarkably, in these conditions, all the infected cells showed C protein accumulation in defined spherical structures (Fig. 1A, right panel). Higher magnification of the images using confocal microscopy revealed that the C protein was organized in a ring-like pattern (Fig. 1A). Co-localization of DENV C with ER or Golgi markers was not observed in these conditions (data not shown). The images of C labeling after DENV infection resembled the distribution of the core protein reported for hepatitis C (HCV), which accumulates on the surface of lipid droplets (LDs) [27]–[29]. To analyze whether DENV C associates to these organelles, infected cells were labeled with antibodies against C and incubated with BODIPY, which stains neutral lipids in LDs. These studies revealed that most of the C protein observed was present around LDs (Fig. 1B). Localization of the C protein surrounding LDs was observed in different DENV infected human cells such as HepG2 and HeLa (Fig. 1B and data not shown). In addition, because DENV is a mosquito borne virus, we examined the localization of C in infected mosquito C6/36 cells. The cytoplasmic localization of C in these cells was also surrounding LDs (Fig. 1B).

To further study the association of C with LDs, sucrose gradients were used to separate the LD fraction by flotation. The presence of C and the adipose differentiation-related protein (ADRP or adipophilin, LD marker) were detected by western blots. A fraction of C was detected together with ADRP in LDs (Fig. 1C). In this fraction the lactate dehydrogenase activity was not detected, indicating lack of cytosolic contamination. The amount of C observed in the LD fraction was lower than that expected according to the co-localization observed with BODIPY (Fig. 1C). It is possible that the viral protein partially dissociates during cell disruption and biochemical fractionation. In order to further analyze the localization of C in the cytoplasm of DENV infected cells, co-localization of C with ADRP was also determined. These studies showed the presence of C and ADRP on LDs (Fig. 1D). Early after infection, we observed single LDs carrying both proteins, C and ADRP. In addition, droplets containing either C or ADRP were also observed.

LDs are ER-derived organelles that contain a core of neutral lipids enclosed by a monolayer of phospholipids and exhibit variable protein content [30]. The metabolism of LDs has attracted considerable attention due to its link with human diseases such as obesity, inflammation, and cancer [31],[32]. LDs are found in different cell types in normal conditions. However, it was noticeable that DENV infection increased the size and the amount of LDs per cell. Quantitative analysis showed a 3-fold increase in the amount of LDs in DENV infected cells as compared with mock infected cells (Fig. 1E). To investigate whether C was the viral factor responsible for the increase in the number of LDs, droplets were enumerated in cells expressing only the C protein. BHK cells were transfected with an expression vector encoding the mature form of C or a control vector. The level of expression of the C protein was slightly higher than that observed in DENV infected cells. Enumeration of droplets indicated that the viral protein increased about 2-fold the amount of LDs per cell (Fig. 1F). The higher increase of LDs observed after DENV infection in respect to that observed in cells expressing only C could be due to the different source of the protein when it is produced from the viral polyprotein. In addition, it is possible that other viral factors or the infection itself affects LD metabolism. Thus, we evaluated the amount of LDs in DENV replicon-expressing BHK cells. In this case, the amount of LDs was not significantly different to that observed in replicon-cured cells (data not shown).

The accumulation of the viral C protein around LDs and the increased number of droplets observed in DENV-infected cells provide the first link between these organelles and DENV replication.

### The mature C protein is targeted to LD in the absence of other viral proteins

**Figure 2:**
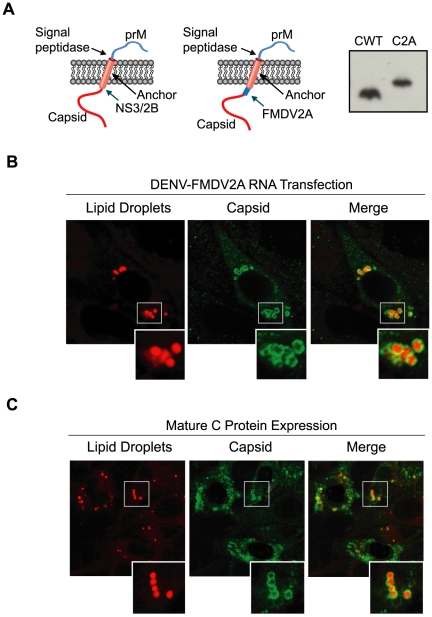
The C protein contains the structural determinants for LD targeting. A. Schematic representation of the topology of the viral C and prM proteins on the ER membrane. The anchor peptide and the cleavage sites of the signal peptidase and viral NS3/2B proteases are indicated. The location of the FMDV2A protease replacing the NS3/2B site is shown in the scheme on the right. The western blot shows expression of the C protein in cytoplasmic extracts of cells transfected with a full length DENV RNA WT (Cwt) or the RNA including the FMDV2A site (C2A). B. The anchor peptide is dispensable for C accumulation on LDs. BHK cells transfected with the DENV-FMDV2A RNA were fixed and probed with antibodies against C and BODIPY to stain neutral lipids in LDs, as indicated on the top. C. Expression of the mature C protein in the absence of other viral components is sufficient for LD targeting. BHK cells were transfected with an expression plasmid that encode the mature form of DENV C protein. Twenty four h post-transfection cells were fixed and probed with anti-C antibodies followed by staining of lipid droplet.

During flavivirus polyprotein synthesis, the C protein is targeted to the ER membrane by the anchor peptide, which is removed by the viral NS3/2B protease in the cytoplasm and the host signal peptidase in the ER lumen (Fig. 2A, left panel). To investigate whether the anchor peptide plays a role in targeting the C protein to LDs, a full-length genomic DENV cDNA was modified to include an artificial FMDV2A cleavage site at the C-terminus of the C protein (DENV-FMDV2A), which would release co-translationally the mature C protein. Transfection of DENV-WT or DENV-FMDV2A RNAs into BHK cells resulted in efficient translation and amplification of viral RNAs (data not shown). Appropriate cleavage of C by the FMDV 2A was demonstrated by Western blot analysis of cytoplasmic extracts obtained at 24 and 48 h post-transfection using anti-C antibodies (Fig. 2A, right panel). As expected, DENV-FMDV2A RNA produced a C protein about 2 kDa larger than the WT protein, corresponding to C plus 19 amino acids of the FMDV2A (Fig. 2A, C2A). Confocal microscopy analysis indicated that the prematurely processed C protein localized almost exclusively around LDs, indicating that the anchor peptide that targets the C protein to ER membranes during polyprotein synthesis is not required for protein C localization on LDs (Fig. 2B).

To determine whether C association to LDs requires other viral components, the mature C protein was expressed using a plasmid under control of the CMV promoter in BHK cells. Cells were analyzed by immunofluorescence using anti-C antibodies and stained with BODIPY at 10, 24 and 48 h post-transfection. Although the level of mature C protein expressed in BHK cells was higher than that observed after DENV infection, most of the expressed C protein also accumulated around LDs (Fig. 2C). This analysis indicates that the mature C protein, in the absence of other viral components, is able to associate to LDs.

### Specific amino acids in the α2 helix are involved in C association to LDs

**Figure 3:**
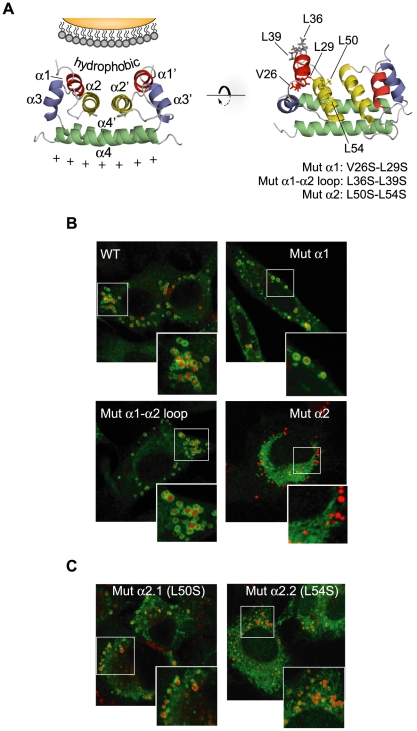
Amino acids within the α2 helix of C are necessary to direct the protein to LDs. A. Ribbon diagram of the dimer structure of DENV C protein [25]. The four α helices (α1 to α4) are indicated in each monomer. The hydrophobic cleft proposed to interact with membranes is also shown. On the right, the location of amino acids that were mutated in the DENV infectious clone is indicated in the structure (Mut α1, Mut α1–α2 loop, and Mut α2). B. Distribution of the C protein and lipid droplets in cells transfected with mutated DENV RNAs. BHK cells transfected with the WT or mutated RNAs containing the substitutions indicated in A were analyzed by immunofluorescence and confocal microscopy. The C protein and lipid droplets were localized by anti-C antibodies (green) and BODIPY (red), respectively. C. Amino acids L50 and L54 are necessary for targeting C to LDs. BHK cells transfected with DENV RNAs carrying the individual substitutions L50S (Mut α2.1) or L54S (Mut α2.2) were used to analyze the localization of the mutated C proteins and LDs as described above.

The molecular basis of C protein association to LDs was then investigated. To this end, we used the model proposed for DENV C interaction with cellular membranes based on the structural information previously obtained by NMR [25]. The model implicates a concave shaped hydrophobic cleft including amino acids of α1 and α2 helices and the connecting loop (Fig. 3A, left panel). We also considered the information provided in previous analysis describing a flavivirus conserved internal hydrophobic region, spanning amino acids 46 to 66 (α2 and α3) in DENV, which was proposed to interact with ER membranes [9]. Amino acids substitutions of residues around the hydrophobic cleft were designed in the context of the full length DENV genome as described in Fig. 3A, and localization of the C protein was followed by confocal microscopy after RNA transfection. Substitutions of uncharged amino acids in α1 helix or in the α1–α2 connecting loop resulted in C proteins that accumulated in LDs, similar to that observed with the WT virus (Fig. 3B). In addition, deletion of the complete α2 helix or substitution of hydrophobic amino acids within α3 resulted in the synthesis of an unstable C protein that was barely detected by immunofluorescence (data not shown). Interestingly, a substitution of the two hydrophobic residues (L50 and L54) within α2 that are facing outwards from the α2−α2′ plane, rendered a C protein that was distributed throughout the cytoplasm without evident association to LDs (Fig. 3B, Mut α2), providing evidence of an important role of these amino acids in C protein-membrane association.

To better define the role of L50 and L54 on C targeting to LDs, we designed the individual mutants L50S (Mut α2.1) and L54S (Mut α2.2). Localization of C after RNA transfection showed a defect in the distribution of these proteins in the cytoplasm when compared with the WT (Fig. 3C). We observed the presence of Mut α2.1 and Mut α2.2 C proteins throughout the cytoplasm; however, in contrast to that observed with the Mut α2, small patches of Mut α2.1 and Mut α2.2 C proteins were detected on LDs (Fig. 3C). These results indicate that both amino acids, L50 and L54, are necessary for proper targeting of C to LDs.

### Mutant α2 retains the ability to bind RNA and to dimerize in solution

**Figure 4:**
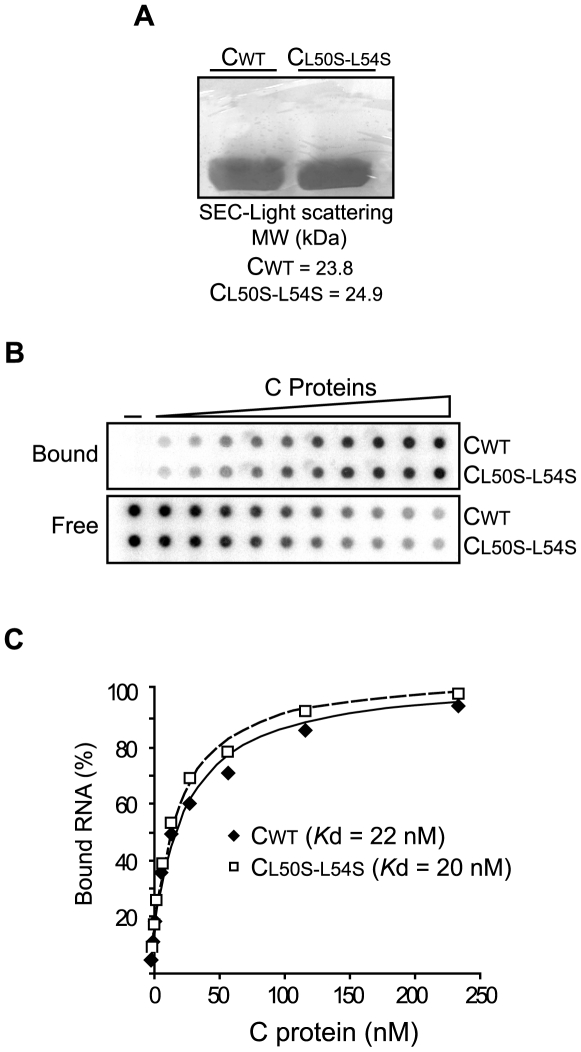
Biochemical properties of recombinant C protein with substitution L50S–L54S. A. High expression levels and dimerization of C_WT_ and C_L50S–L54S_. SDS-PAGE stained with coomassie blue showing similar expression levels of the recombinant proteins. The molecular mass obtained by size exclusion chromatography (SEC) and light scattering for both proteins are indicated. B. Interaction of C_WT_ and C_L50S–L54S_ with the DENV 5′UTR RNA probe monitored by filter binding assay. Uniformly ^32^P labeled RNA (0.1 nM) was incubated with increasing concentrations of the respective C protein. Bound indicates RNA-protein complexes retained in the nitrocellulose membrane and free denotes the unbound probes retained in the nylon membrane. The RNA probes bound and free in each membrane were visualized by PhosphoImaging. C. Quantification of the percentage of RNA probe bound was plotted as a function of C concentration and fitted using equation 1 (see Materials and methods). The dissociation constants K*ds* are indicated inside the plot.

To investigate whether the mutation L50S–L54S alters C protein folding, dimerization, or RNA binding, biochemical properties of the recombinant proteins were analyzed. The mature WT and mutated C proteins were cloned in an expression vector in the absence of a tag. Purification was performed by heparin columns and gel filtration. Expression and purification of the C_L50SL54S_ mutant were indistinguishable from the WT protein (Fig. 4A). The oligomerization state of the proteins was determined by size exclusion chromatography and light scattering. Single picks corresponding to molecular weights of 23.8 and 24.9 kDa were obtained for the C_WT_ and the C_L50SL54S_ respectively, which are consistent with dimer formation.

To determine whether the mutation could interfere with the ability of the C protein to bind RNA, mobility shift and filter binding assays were performed to estimate the dissociation constants. A radiolabeled RNA was used for titration with different concentrations of C_WT_ or C_L50SL54S_. The dissociation constants were not significantly different, 22 nM and 20 nM for the WT and the mutant, respectively (Fig. 4B and 4C). The results indicate that the L50S–L54S mutation introduced in the C protein did not alter protein folding or other known properties of the protein.

### Association of C to LDs is necessary for DENV replication

**Figure 5:**
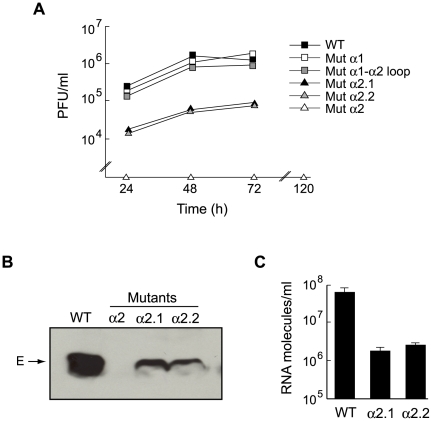
Targeting the C protein to LDs is necessary for DENV production. A. The media of BHK cells transfected with DENV RNA WT or mutants (Mut α1, Mut α1–α2 loop, Mut α2, Mut α2.1, and Mut α2.2) were collected as a function of time post-transfection and used to quantify the amount of infectious particles by plaque assay in BHK cells. The plot indicates the plaque forming units per ml at different times post-transfection. B. The secreted enveloped protein E was analyzed in the supernatant of transfected cells by western blot as previously described [33]. C. BHK cells were infected with a multiplicity of infection of 0.1 of WT, Mut α2.1, and Mut α2.2 viruses. The viral RNA was quantified by real time RT-PCR in the media obtained 24 h post-infection.

To investigate the effect of mutating C on DENV replication, cells were transfected with WT or mutant RNAs that produce stable C proteins (Mut α1, Mut α1–α2 loop, Mut α2, Mut α2.1, and Mut α2.2). Viral replication in transfected cells was evaluated by immunofluorescence as a function of time and by assessing the production of infectious viral particles by plaque assay. Mut α1 and Mut α1–α2 loop produced titers similar to the WT at 24, 48 and 72 h (Fig. 5A). After 96 h the titers decreased due to extensive cytopathic effect and death of the transfected cells. In contrast, the titers for Mut α2.1 and Mut α2.2 were about two orders of magnitude lower than that for the parental virus. In addition, no viral particles were detected in the supernatants of cells transfected with Mut α2 up to 5 days post-transfection (Fig. 5A). Furthermore, the immunofluorescence assays indicated that while the WT, Mut α1, and Mut α1–α2 loop showed the complete monolayer antigen-positive for DENV at day 3, Mut α2.1 and Mut α2.2 showed a propagation delay, and no viral propagation was detected in cells transfected with Mut α2 until day 15 (data not shown). The results indicate that mutations that alter C targeting to LDs produced defects in viral replication.

To investigate whether the viruses carrying the mutations in the α2 helix produced viral particles that were not infectious, we determined the presence of the viral envelope (E) protein in the media. Western blot analysis indicated that the amount of the E protein released from cells transfected with Mut α2.1 and α2.2 was less than 5% of that observed with the WT (Fig. 5B). In addition, the E protein was undetectable in the media of cells transfected with Mut α2 RNA. Moreover, viral RNA was quantified in the media of cells infected with WT, Mut α2.1, and α2.2 using real time RT-PCR (Fig. 5C). The amount of viral RNA detected for both mutants was about two logs lower than that for the parental virus, which correlated with the amount of infectious particles produced in Fig. 5A. These results indicate that the mutations in the α2 helix of the C protein impair the production of DENV particles.

### Dissecting cis-acting RNA replication signals from the C coding sequence

**Figure 6:**
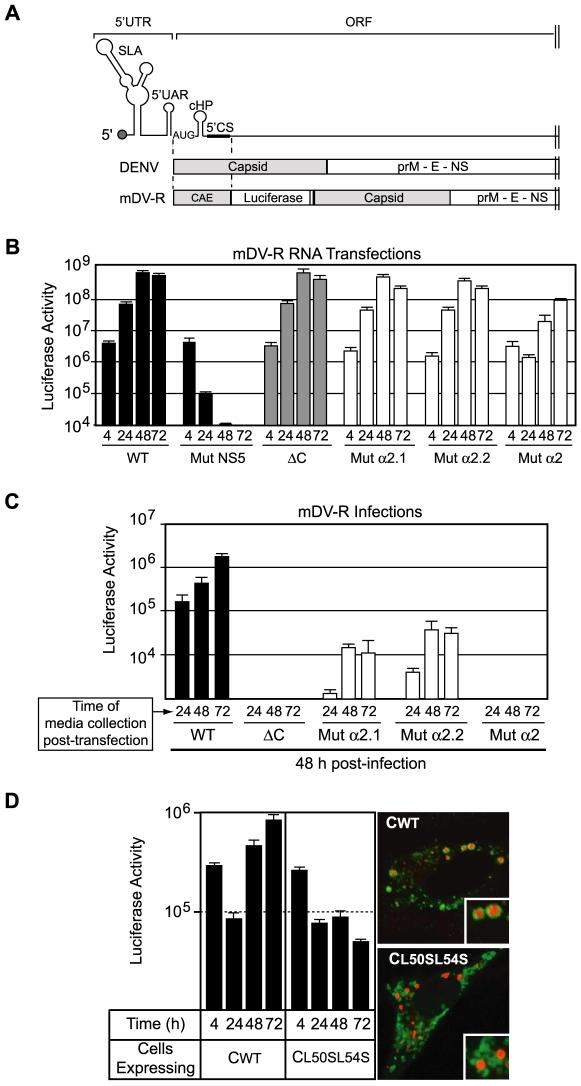
A new reporter virus that allows dissociation of cis-acting RNA elements from the capsid coding region confirms a role of L50 and L54 in DENV particle formation. A. Construction of a novel monocistronic DENV reporter system. At the top, schematic representation of the cis-acting replication elements located at the 5′ end of the DENV genome. The promoter stem-loop A (SLA), the cyclization sequence upstream of the AUG (5′UAR), the replication element cHP, and the cyclization sequence 5′CS are indicated. In the middle, the corresponding region of DENV polyprotein is shown. At the bottom, a schematic representation of the monocistronic DENV reporter construct (mDV-R) showing the duplication of the cis-acting elements (CAE) and the location of the luciferase and the viral proteins. B. Translation and replication of mutant mDV-R RNAs. BHK cells were transfected with DENV RNAs corresponding to the mDV-R WT, Mut ΔC with the complete deletion of C coding sequence, Mut α2.1, Mut α2.2, Mut α2, and Mut NS5, which carries a mutation in the catalytic GDD motif of the viral polymerase. Luciferase activity was measured as a function of time for each RNA as indicated at the bottom. C. Mutations in the α2 helix of the C protein impair viral particle formation. The media of the transfected cells from the experiment shown in B was collected at the indicated times and used to infect fresh cells. Luciferase activity was measured 48 h post-infection for each virus as indicated at the bottom. D. A matured form of C_L50SL54S_ protein expressed in BHK cells decreased the levels of DENV RNA synthesis. Immunofluorescence of BHK cells expressing the DENV C_WT_ or C_L50SL54S_ probed with anti C (green) and stained with Bodipy (red) for lipid droplets are shown in the right panel. The cells transfected with DV-R RNA WT were used to measure luciferase activity as a function of time, as indicated in the left panel.

We have recently developed a DENV reporter system to evaluate each step of DENV replication [33]. To further characterize the defect of the DENV C mutants, we introduced the substitutions in the reporter virus (DV-R). Controls and mutated viral RNAs were transfected in BHK cells and luciferase activity was monitored as a function of time as previously reported [33]. Unexpectedly, transfection of Mut α2 DV-R showed a delayed increase in luciferase activity during viral RNA synthesis (data not shown). Because flavivirus structural proteins do not participate in viral RNA amplification [34],[35], this observation was puzzling. It is possible that the substitution introduced in the α2 helix alters RNA structures present in the C coding sequence that have been previously reported to be involved in genome cyclization and RNA amplification [3]. In fact, the presence of overlapping signals in the viral genome has been a limitation in studying the effect of mutations in the N-terminus of C on viral encapsidation. Thus, to properly analyze the defects in replication of DENV C mutants, we designed a new DENV reporter system dissociating the cis-acting signals from the C coding region. To this end, we introduced a duplication of the first 104 nucleotides of the C coding region, called here the cis-acting element CAE (including the previously described cHP and the cyclization sequence 5′CS) [36]–[38]. The CAE was fused to the luciferase coding region followed by the complete DENV ORF (Fig. 6A, monocistronic DENV reporter, mDV-R). Between the luciferase and the DENV structural proteins an FMDV2A protease was introduced to ensure the release of the reporter protein. In summary, the new reporter DENV contained a physical separation of the CAE sequences and the C coding region. Transfection of the mDV-R RNA resulted in efficient viral replication and production of infectious viral particles (Fig. 6B and C, WT).

To investigate the replication of mutants in the α2 helix that impair LD association without altering the cis-acting RNA elements, Mut α2, Mut α2.1, and Mut α2.2 were introduced in the mDV-R. The RNAs corresponding to the mDV-R WT, the three mutants in the α2 helix, the propagation impaired mutant containing the complete deletion of C coding sequence (Mut ΔC), or the replication impaired mutant carrying a substitution in the polymerase NS5 (Mut NS5), were transfected into BHK cells (Fig. 6B). The Mut ΔC mDV-R showed luciferase levels at 24 and 48 h post-transfection that were indistinguishable from the WT mDV-R levels, confirming that the C protein is dispensable for RNA synthesis and indicating that the duplication of the CAE was fully functional (Fig. 6B, compare Mut ΔC with the positive and negative controls, WT and Mut NS5, respectively). Similarly, Mut α2.1 and Mut α2.2 translated and replicated the RNA efficiently. In contrast, while the Mut α2 RNA was translated as the parental RNA (see luciferase activity at 4 h post-transfection), the luciferase levels detected at 24 and 48 h were reduced about 40 fold in respect to the WT control (Fig. 6B). These results indicate that while deletion of the complete C protein or the individual mutations L50S and L54S did not affect DENV RNA synthesis, the more drastic change that included both substitutions did, and this effect was not due to alteration of the cis-acting elements.

To analyze the ability of the mutants in the C protein to produce reporter infectious particles, we collected the supernatants of the transfected cells as a function of time and used them to infect fresh BHK cells. As expected, the luciferase activity in cells infected with the media obtained from cells transfected with Mut ΔC was undetectable (Fig. 6C). Similarly, the Mut α2 failed to produce viral particles. After infection with the media of cells transfected with Mut α2.1 or Mut α2.2, between 50 and 200 fold lower luciferase activity than that with WT mDV-R was observed. These results confirm a direct role of amino acids L50 and L54 on viral particle formation.

The decreased level of RNA amplification of Mut α2 presented in Fig. 6B was unexplained; thus, we decided to further analyze this observation. Knowing that the C protein has high affinity for RNA molecules, a plausible explanation could be that a mistargeted C protein, which accumulates in the cytoplasm, prematurely binds the viral RNA or interacts with other factor involved in viral RNA replication. To analyze this possibility, we studied the RNA synthesis of WT DENV in cells producing the WT or mutated C proteins in trans. BHK cells expressing a mature form of C_WT_ or C_L50SL54S_ were transfected with the WT reporter DENV RNA, and luciferase activity was monitored as a function of time. Over-expression of C_WT_ or C_L50SL54S_ proteins was not toxic for BHK cells as determined by MTS assays. Cells expressing C_WT_ showed accumulation of the viral protein in LDs, while the ones expressing C_L50SL54S_ showed a cytoplasmic distribution without a significant accumulation in LDs (Fig. 6D, right panel). Luciferase activity was determined in cells at 4, 24, 48 and 72 h post-transfection (Fig. 6D). Cells expressing the C_WT_ showed luciferase levels at 48 and 72 h about 10 and 30 fold higher, respectively, than those in cells expressing the C_L50SL54S_. These results suggest that the mutated protein expressed in trans was able to decrease the level of viral RNA amplification.

Taken together, the new reporter DENV allowed us to dissociate the processes of RNA replication and encapsidation, demonstrated that C is dispensable for RNA synthesis, and confirmed an important role of amino acids L50 and L54 in viral particle formation. In addition, the results suggest that a mislocalized C protein could interfere with viral RNA synthesis, providing evidence for a possible role of LDs in coordinating different viral processes.

### LDs as target for DENV inhibition

**Figure 7:**
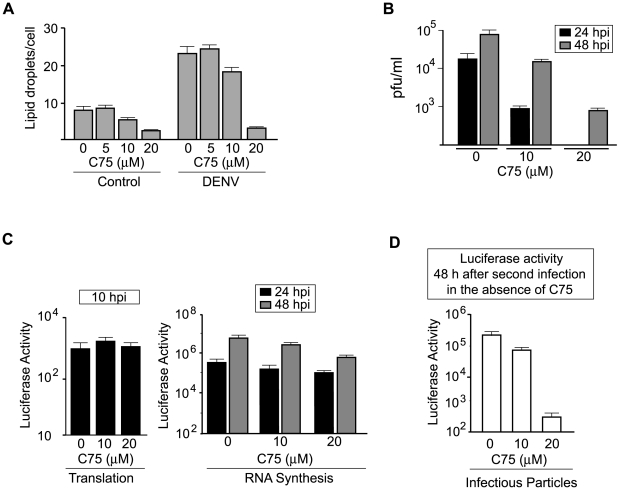
Pharmacological inhibition of lipid droplets accumulation impairs DENV replication. A. Effect of C75 on the amount of lipid droplets in BHK cells. The amount of lipid droplets was quantified in BHK cells treated with different concentrations of C75. Control or DENV infected BHK cells were used. B. Inhibition of DENV replication in cells treated with C75. The amount of infectious viral particles produced at 24 and 48 h post-infection in BHK cells were evaluated by plaque assays in control or C75 treated cells as indicated. Error bars indicate the SD of three independent experiments. C. Effect of C75 on each step of the replication of the mDV-R. Viral stocks of the reporter mDV-R were used to infect BHK cells in the presence and absence C75. Luciferase activity was evaluated at 10 h post-infection to evaluate entry and translation (left panel), and at 24 and 48 h to evaluate RNA synthesis (right panel). D. The production of infectious viral particles produced in the experiment described in C was evaluated by infecting fresh BHK cells in the absence of the inhibitor, and assessing the luciferase activity 48 h after infection.

Here, we found that targeting C protein to LDs is necessary for DENV particles formation. In addition, we observed that viral infection increases the amount of LDs. Based on these findings, we hypothesized that interfering with LDs formation/metabolism could be a means for antiviral intervention. To prove this idea, we used a fatty acid synthase inhibitor (C75) that was previously designed for obesity control [39]–[41]. It has been reported that this drug reduces the amount of LDs in the cell and inhibits pre-adipocyte differentiation. First, we analyzed the effect of C75 on the amount of LDs in DENV-infected and non-infected cells. The concentration of drug used was determined to be non-toxic for BHK cells (data not shown). Quantitative analyses of LDs in BHK cells showed that concentrations between 10 and 20 µM of drug decreased the amount of LD in DENV-infected and mock-infected cells (Fig. 7A). To determine the effect of C75 on viral replication, cells were treated with 10 and 20 µM of compound, infected with DENV2 using a multiplicity of infection of 1, and viral titers were determined at 24 and 48 h post-infection by plaque assay (Fig. 7B). Using 20 µM of C75, a drop in two orders of magnitude in the viral titer at 48 h and complete inhibition of viral replication at 24 h were observed. Similar results were obtained when C75 treated HepG2 cells were infected with DENV (data not shown). To determine how the drug affects each step of viral replication, the reporter DENV was used. Luciferase activity was measured in extracts of BHK cells infected with mDV-R in the presence or absence of C75. At 10 h post-infection the luciferase levels were unaffected by the inhibitor, suggesting that the drug was not interfering with viral entry or translation (Fig. 7C, left panel). At 24 and 48 h post-infection a reduction of luciferase levels of about 4-fold was observed, which corresponds to a decrease in RNA amplification. To investigate the effect of the drug on infectious viral particle formation, the media from cells subjected to each treatment was collected 48 h after infection and used to infect fresh cells in the absence of C75. At this time, an inhibition of more than 1000-fold was observed, indicating a profound effect of C75 on viral particle production (Fig. 7D). These results indicate that altering the LD metabolism can be a means to block DENV replication.

## Discussion

Genome packaging is one of the most obscure steps of the flavivirus life cycle. Here, we provide the first evidence linking DENV particle formation with ER derived LDs. We found that DENV infected cells accumulate the C protein around LDs and this localization is crucial for infectious particle formation. Specific hydrophobic amino acids were identified as key determinants for LD association. In addition, we developed a new genetic tool to exclude cis-acting RNA replication signals from the C coding sequence. Using this system, we found that mislocalization of a mutated C protein interferes with DENV RNA synthesis. Our studies support the idea that DENV exploits LDs for multiple purposes during DENV replication. Furthermore, relevant to the urgent need for antiviral strategies against DENV, we report that pharmacologic alteration of LD metabolism also inhibits DENV replication in cell culture.

### Structural features of *Flaviviridae* C proteins and their association to LD

*Flavivirus* is one of the three genera of the *Flaviviridae* family together with the *Hepaci-* and *Pestivirus* [2]. The C proteins of the three genera do not exhibit significant sequence homology or common domain organization. However, they are all dimeric, basic proteins with an overall helical fold, responsible for genome packaging. In addition, a recent report has suggested a common RNA chaperone activity for these C proteins [42]. Hepacivirus mature core proteins are about 170 amino acids in length and consist of two domains, a highly basic N-terminal domain (D1) and a hydrophobic C-terminal domain (D2) [43]. In contrast, pesti- and flavivirus C proteins are shorter, between 90 to 100 residues, lacking a D2 domain. Compelling evidence has been accumulated in recent years supporting the idea that HCV particle formation requires C protein association to LDs, and that the D2 domain is responsible for targeting C to this organelle [28], [29], [44]–[49]. Because the flavivirus C proteins lack a D2 domain, an association of DENV C protein to LDs was unexpected.

Using DENV-infected cells, we found that the C protein accumulated on LDs. Hydrophobic residues in the α2 helix of DENV C were defined as important determinants for LD association and viral particle formation. In contrast, mutations of uncharged residues in α1 helix or in the connecting loop between α1 and α2 helices did not alter LD association or viral propagation. The importance of an internal hydrophobic region including the α2 helix was originally described in DENV4, and more recently was reported to be necessary for efficient propagation of different flaviviruses [9], [50]–[52]. A recent study using WNV reported that deletions within the most hydrophobic section of α2 helix (LALLAFF) impaired viral propagation [53]. However, pseudorevertants with extended deletions of C from amino acid 40 to 76 were recovered in culture. These results indicated that a large deletion of about 36 amino acids was better tolerated than 4–7 amino acid deletions in the hydrophobic region, suggesting that a short version of the C protein could form nucleocapsids by an alternative mechanism. A remarkable functional flexibility of the C protein was observed in TBEV, in which deletions from 19 to 30 residues were rescued by second site mutations increasing the hydrophobicity of the protein [51],[54]. Studies using a YF replicon trans-packaging system demonstrated that large deletions in the N and C terminal regions of protein C were tolerated [50]. In the same report, using a YFV infectious clone, it was shown that the C protein with deletions of the α1 helix resulted in small plaque phenotypes, while deletions including α1 and α2 were lethal. Using DENV, we observed that mutations of amino acids L50 or L54 within α2 helix of C greatly decrease viral particle formation. These results are in agreement with a previous study, in which a deletion of residues 42 to 59 in DENV C protein in α2 impaired viral propagation [52].

According to our findings, hydrophobic amino acids within the α2 helix in the center of DENV C protein would function as the hepacivirus C- terminus D2 domain in targeting the protein to LDs. We conclude that hepaci- and flaviviruses use distinct structural features of the C protein for subcellular localization, suggesting a convergent evolution of these viral proteins. It remains to be examined whether the pestivirus C proteins also accumulate on LDs.

### Biological significance of LD in DENV replication

Viral infection could modulate a range of host cell functions and usurp the cellular organization to facilitate viral spread. Although viral translation, RNA amplification, and encapsidation must be temporally and spatially regulated in the cytoplasm of the infected cell, the mechanisms by which flaviviruses coordinate these processes are still unclear. Here, we constructed a new genetic tool to dissociate overlapping signals for DENV RNA replication and encapsidation (mDVR, Fig. 6A). This tool allowed us to confirm that complete deletion of the C protein did not alter viral RNA translation or RNA synthesis. The substitution L50S or L54S, which altered C targeting to LDs, resulted in viruses that translated and replicated the RNA efficiently but had defects in infectious particle production (Fig. 6B and C). These viruses released reduced amounts of viral E protein and viral RNA, supporting the idea that C association to LDs is necessary for viral particle formation (Fig. 5). The double mutant (L50S+L54S), which abolished protein association to LDs and impaired viral particle production, was also found to delay amplification of viral RNA (Fig. 6B, Mut α2). It is possible that accumulation of this mutated C protein in the cytoplasm could interact with the viral RNA and interfere with genome amplification. A biological role of LDs as transient depots to store or sequester proteins that are in temporary excess has been previously reported [55]. Sequestration of histones on LDs that are released during development has been demonstrated [55]. Therefore, similarly to that observed with histones, LDs could temporally control viral processes by regulating the availability of the highly basic C protein in the cytoplasm of infected cells. Interestingly, localization of C on LDs was also observed in mosquito cells, suggesting a conserved function of these organelles in viral replication in different hosts.

The place and the mechanism by which the C protein recruits the viral RNA to form the nucleocapsid in the infected cell are still unclear. Because a dynamic shift of proteins and lipids between the ER and the LDs has been reported (for review see [30]), it is possible that C is stored on LDs early during infection to be then mobilized to the ER membrane for particle morphogenesis. Alternatively, the genomic RNA could interact with C on the surface of LDs to form the nucleocapsids, which could be then transferred to the ER membrane for new viral particles formation.

We observed that DENV infection increases the amount of LDs per cell (Fig. 1C). A recent functional genomic screen revealed a number of genes involved in LD formation and the regulation of their number, morphology, and distribution in the cell [56]. Thus, it will be important to investigate how DENV alters these pathways to increase the formation of new LDs or change the half life of the already existing ones. In addition, it will be interesting to examine the effect of the C protein on the enzymatic activities involved in lipid metabolism that have been found associated to LDs. In the case of HCV, interaction of the C protein with LDs was linked to increased lipid accumulation and hepatic steatosis in transgenic mice [57],[58]. Because liver steatosis has been also observed in DENV-infected mice and fatal cases of DHF in humans [59],[60], it is relevant to investigate a possible correlation between LD accumulation in infected tissues and DENV pathogenesis.

The properties of LDs have attracted considerable interest because of the link between enhanced fat storage and human diseases such as obesity, inflammation, and cancer. In recent years different compounds that affect the accumulation and metabolism of LDs have been developed [61]–[63]. Here, we found that a fatty acid synthase inhibitor (C75) that decreased the amount of LDs in DENV-infected and uninfected cells, also inhibited dengue replication 100 to 1000 fold (Fig. 7B). Using a luciferase DENV reporter system, we observed that C75 did not alter viral entry or viral translation. Although the most pronounced inhibition was observed in the production of infectious viral particle, a low but significant reduction of RNA synthesis was also detected. This effect could be due to alteration of the metabolism of lipids, which are components of the replication complexes. In addition, the decreased amount of LDs observed with C75 could account for the large reduction in viral particles produced.

Currently, dengue fever and dengue hemorrhagic fever are a tremendous social and economic burden on the world population. We believe that uncovering molecular details of the DENV life cycle and understanding the host pathogen interaction will aid the search for novel anti-dengue strategies.

## Materials and Methods

### Ethics statement

Research involving animals was approved by the IACUC of the Leloir Institute fully complying with the National Institute of Health (NIH, USA) guidelines.

### Cells and viruses

Baby hamster kidney cells (BHK-21) were cultured in minimum essential medium alpha supplemented with 10% fetal bovine serum, 100 U/ml penicillin, 100 µg/ml streptomycin. Human hepatocellular liver carcinoma cell line (HepG2) was cultured in minimum essential medium supplemented with 10% fetal bovine serum, 100 U/ml penicillin, 100 µg/ml streptomycin and 0.01% sodium pyruvate. C6/36 HT mosquito cells from *A. albopictus*, adapted to grow at 33°C, were cultured in L-15 Medium (Leibovitz) supplemented with 0.3% tryptose phosphate broth, 0.02% glutamine, 1% MEM non-essential amino acids solution and 5% fetal bovine serum. Stocks of DENV serotype 2 16681 were prepared in mosquito C6/36 cells and used to infect the different cell lines as indicated in each case.

### Construction of recombinant DENVs

The desired mutations were introduced in a DENV type 2 cDNA clone [64] (GenBank accession number U87411) by replacing the *Sac*I-*Sph*I fragment of the WT plasmid with the respective fragment derived from an overlapping PCR. The sequence of the oligonucleotides used as primers for all the PCR reactions are listed in Table 1. To generate the plasmids carrying the mutations L50S, L54S, L50S–L54S, L36S–L39S and V26S–L29S, common outside primers 101 and 239 were used. Mutation L50S was generated using the inside primers 1035 and 1036, mutation L54S using primers 1037 and 1038, mutation L50S–L54S using primers 833 and 832, mutation L36S–L39S with primers 1050 and 1049, and mutation V26S–L29S with primers 1054 and 1053.

**Table 1:** Sequence of oligonucleotides.

#	Sequence
7	GTGGGTTCGAAAGTGAGAATCTCTTTGTCAGCT
101	TCCAGACTTTACGAAACACG
239	TCTGTGAT GGAACTCTGTGG
241	TTTGACATTCCTATGCAACG
273	GAATTCGAGCTCACGCGTAAATTTAATACGACTCACTATAAGTTGTTAGTCTACGTGG
487	ATCTCTGCCATGGGTAATAACCAACGGAAAAAGGCG
489	TGCAGAGGATCCTCATTATCTGCGTCTCCTATTCAAGATG
516	GACGTCTCCCGCAAGCTTGAGAAGGTCAAAATTCAACAGCTGTTGTTCATTTTTGAGAACTCGC
517	CTTCTCAAGCTTGCGGGAGACGTCGAGTCCAACCCTGGGCCAATGAATAACCAACGGAAAAAGGCG
595	GTGATGATTTACCAAAAATGTTTATTGAATCGG
832	GGAAACGTGAGAACGCCACTGAGGCCATGAACAGTTTTAATGG
833	CATGGCCTCAGTGGCGTTCTCACGTTTCCTA ACAATCCCACC
947	ATCTCTCTTAAGATGAATAACCAACGGAAAAAGG
1030	GGCAAGCTTGAGTAAATCAAAATTTAGGAGCTGTTGTTCATTTTTGAGAACC
1031	TTCTCAAAAATGAACAACAGCTCCTAAATTTTGATTT ACTCAAGCTTGCCGGC
1035	GGAAACGAAGGAACGCCACTGAGGCCATGAACAGTTTTAATGG
1036	CATGGCCTCAGTGGCGTTCCTTCGTTTCCTAACAATCCCACC
1037	GGAAACGTGAGAACGCCACCAGGGCCATGAACAGTTTTAATGG
1038	CATGGCCCTGGTGGCGTTCTCACGTTTCCTAACAATCCCACC
1049	CGTCCCTGTGACATTCCCGATGAGAATCTCTTTGTCAG
1050	GAGATTCTCATCGGGAATGTCACAGGGACGAGGACC
1054	CCGCGTGTCGACTTCACAACAGTCAACAAAGAGATTCTCACTTGG
1053	CTCTTTGTTGACTGTTGTGAAGTCGACACGCGGTTTCTCTCGC

Bicistronic dengue virus reporter constructs (DV-R) containing the reporter Renilla luciferase was previously described [33]. The monocistronic DENV reporter construct was build using a previously described plasmid pD2/IC*Afl*II [35] including an additional *Not*I restriction site at nucleotide 244 (pD2/IC*Afl*II*-Not*I). To facilitate insertion of the *Renilla luciferase* gene (R*luc*), we generated an intermediate plasmid derived from pRL-CMV (Promega). Using unique *Sac*I and *BstB*I restriction sites, we introduced the complete DENV 5′UTR followed by the first 104 nucleotides of the coding sequence of C, using primers 101 and 7. The resulting plasmid was used to introduce downstream of R*luc* the FMDV2A protease coding sequence (QLLNFDLLKLAGDVESNPGP) fused to the capsid protein. The fragment carrying FMDV2A fused to DENV sequences was generated by overlapping PCR using for the first PCR primers 273 and 516, and for the second PCR primers 517 and 241. The overlapping PCR product was digested with *Sac*I-*Not*I restriction enzymes and introduced into homologous restriction sites within pD2/IC*Afl*II*-Not*I. To generate mDV-R Mut L50S, mDV-R Mut L54S, and mDV-R Mut L50S–L54S an overlapping PCR was performed with the common primers 595 and 239. The sense and antisense primers used to generate each of the mutations were the same as described above. For mutant mDV-R ΔC, a fragment carrying the deletion of mature C protein was generated by overlapping PCR using the following primers: PCR1 primer sense 595 and primer antisense 1030; and PCR2 primer sense 1031 and primer antisense 239. The overlapping PCR product was cloned into the mDV-R cDNA using the unique restriction sites *Sac*I-*Sph*I.

### RNA transcription, transfection, and viral recovery

Wild-type (WT) or mutant DENV plasmids were linearized with *Xba*I and used as templates for T7 RNA polymerase transcription in the presence of m7GpppA cap analog. RNA transcripts (5 µg) were transfected with Lipofectamine 2000 (Invitrogen) into BHK-21 or HepG2 cells grown in 60-mm-diameter tissue culture dishes. Supernatants were harvested at the indicated times post-transfection and used to quantify infectious DENV particles by plaque assays as previously described [35]. Quantification of viral RNA was performed by real time RT-PCR using TaqMan technology as previously described [35].

### Immunofluorescence assay

BHK-21, HepG2, and C6/36 cells were seeded into 24-well plates containing glass coverslips. Twenty four hours after, they were infected with a DENV2 stock using a multiplicity of infection of 10. At the indicated times the coverslips were removed and the cells were fixed in paraformaldehyde 4%, sucrose 4%, PBS pH 7.4 at room temperature for 20 minutes. Alternatively, they were fixed in methanol for 20 minutes at −20°C. Cells were then permeated with 0.1% Triton X-100 for 4 minutes at room temperature. Rabbit polyclonal antibodies against C were obtained in our laboratory as describe below. A 1∶1000 dilution of this anti-C antibody in PBS–0.2% gelatin was used. Goat anti-rabbit IgG Cy3 conjugated (Jackson Immuno Research) were used at 1∶500 dilution. For lipid droplets staining cells were incubated with BODIPY 493/503 (4,4-difluoro 1,3,5,7,8 pentamethyl 4-bora 3a,4a-diaza-s-indacene) (Molecular Probes) at 1∶500 dilution, 1 µM. For detection of ADRP, a commercial mouse monoclonal antibody (ARP American Research Products, Inc) was used 1/100 in PBS-gelatine. Cy5 AffiniPure Donkey Anti-mouse IgG antibody (Jackson ImmunoReserch) was used 1/500 in PBS-gelatine. Cells were mounted on glass slides and images were obtained with a Zeiss axioplant confocal microscopy. To maintain the consistency of the green color for the C protein, the color of BODIPY was changed to red. For immunofluorescence of transfected cells, the procedure was the same as the one described for infections.

### Purification of recombinant C protein in *E. coli* and production of antibodies

The coding sequences of the mature C protein (amino acids 1–100) were obtained by PCR from the DENV type 2 cDNA clone [64] using the sense primer 487 carrying the restriction site *Nco*I and the antisense primer 489 with the restriction site *BamH*I. The PCR product was digested and cloned into the expression vector pET-15b (Novagen). Protein expression was performed in the *E. coli* strain BL21 Rosetta (DE3)pLysS (Novagen). The bacterial culture was grown at 37°C until OD_600_ = 1, induced with 1 mM IPTG and incubated at 18°C overnight. C protein from soluble fraction was first purified using heparin affinity chromatography, eluted with a gradient from 0.2 M to 2 M of NaCl in 50 mM NaH_2_PO_4_ (pH 7.5). Fractions containing the protein were collected and further purified by size exclusion chromatography using a Superdex 75 column (GE Healthcare). Highly purified fractions of C protein were aliquoted and stored at −70°C in eluted buffer containing 200 mM NaH_2_PO_4_ (pH 6) and 500 mM NaCl. Polyclonal antibodies were obtained by inoculating rabbits three times with 0.2 mg of the purified C protein with Freund's adjuvant (SIGMA). Four days before sacrificing the animals, a booster of C without the adjuvant was injected. The antibodies obtained were evaluated for specificity using western blots and ELISA employing infected and non-infected BHK cell extracts and supernatants.

### Eukaryotic expression of mature C protein

The coding sequences of the mature C protein (amino acids 1 to 100) derived from DENV type 2 were obtained by PCR using the sense primer 947 carrying the restriction site *Afl*II and the antisense primer 489 with the restriction site *BamH*I. The PCR product was digested and cloned in the eukaryotic expression plasmid pcDNA6/V5-HisB (Invitrogen). Purified plasmid (2 µg) was transfected with Lipofectamine 2000 (Invitrogen) into BHK-21 cells grown in 24-well plates containing a 1-cm^2^ coverslip. At different time points after transfection the coverslips were fixed and directly used for IFA.

### Lipid droplet counting

Cells were fixed as described for the immunofluorescence assay and then treated as follows: rinsed in 0.1 M cacodylate buffer, incubated with 1.5% OsO_4_ (30 min), rinsed in H_2_O, immersed in 1.0% thiocarbohydrazide (5 min), rinsed in 0.1 M cacodylate buffer, incubated in 1.5% OsO_4_ (3 min), rinsed in distilled water, and then dried for further analyses. The morphology of fixed cells was observed, and lipid droplets were enumerated by light microscopy with ×100 objective lens. The total amount of lipid droplets was counted in 50 consecutive cells. For each determination the experiment was done in triplicates.

### Isolation of lipid droplets by subcellular fractionation

Lipid droplets were isolated by sucrose gradients as we previously described [41]. Briefly, DENV infected BHK cells in 20 mM Tris, 1 mM EDTA, 1 mM EGTA, 100 mM KCl buffer (pH 7.4) containing a protease inhibitors cocktail were disrupted by nitrogen cavitation at 700ψ for 5 min at 4°C and collected in an equal volume of buffer containing 1.08 M sucrose. The homogenates were centrifuged to remove the nucleus and the supernatant were overlaid with 2 ml each of 0.27 M sucrose buffer, 0.13 M sucrose buffer, and top buffer (25 mM Tris HCl, 1 mM EDTA, and 1 mM EGTA). The gradient was centrifuged at 250,000 g 1 h at 4°C. The fractions collected from the top contained LD, cytosol, microsomal fraction, and pellet. Proteins from these fractions were precipitated overnight with TCA, washed with cold acetone, and analyzed by western blot using anti-C and anti-ADRP (guinea pig anti- ADRP polyclonal antibodies, Research Diagnostics Inc., Flanders, NJ). The activity of lactate dehydrogenase (LDH) was measured using the CytoTox 96 kit (Promega) to discard cytosolic contamination in the LD fraction.

### RNA-binding assays

The interaction of the C protein with RNA was analyzed by filter-binding assays (FBA). Uniformly ^32^P-labeled RNA probe corresponding to the viral 5′ terminal region (nucleotides 1–160) was obtained by in vitro transcription using T7 RNA polymerase and purified on 5% poly-acrylamide gels–6 M urea. The binding reactions contained 50 mM NaH_2_PO_4_ (pH 6), 150 mM NaCl, 0.02% tween 20, 0.1 nM ^32^P-labeled probe, and increasing concentrations of C protein (0, 3.75, 7.5, 15, 30, 60, 125, 250, 500, and 1000 nM). For FBA, Nitrocellulose (Protran BA 85, Whatman-Schleider& Schuell) and Hybond N+ nylon (Amersham Bioscience) membranes were pre-soaked in binding buffer 50 mM NaH_2_PO_4_ (pH 6), 150 mM NaCl, 0.02% tween 20 and assembled in a dot-blot apparatus. A 20-µL aliquot of each protein–RNA mixture was applied to the filters and rinsed with 100 µL of binding buffer. Membranes were air-dried and visualized by PhosphoImaging analysis. The macroscopic binding constants were estimated by nonlinear regression (Sigma Plot), fitting *Equation 1*: Bound % = Boundmax·[Prot]/(*K*d+[Prot]), where Bound % is the percentage of bound RNA, Boundmax is the maximal percentage of RNA competent for binding, [Prot] is the concentration of purified C protein, and *K*d is the apparent dissociation constant.

### Determination of C protein molecular weight by Static Light Scattering (SLS)

The average molecular weight (MW) of the proteins was determined on a Precision Detector PD2010 light-scattering instrument tandemly connected to an FPLC system and a LKB 2142 differential refractometer. Five hundred µl of C protein (1 mg/ml) were loaded on a Superdex 75 HR 10/30 (24 ml) column, size exclusion was performed at 0.4 mL/min with a running buffer of 200 mM NaH_2_PO_4_ (pH 6.0) and 500 mM NaCl. The 90° light scattering, refractive index, and absorbance of the eluting material were recorded on a PC computer and analyzed with the Discovery32 software supplied by Precision Detectors. The 90° light scattering detector was calibrated using BSA as a standard.

### Studies with the inhibitor C75

The compound C75, a fatty acid synthase (FAS) inhibitor, was purchased from Cayman chemicals. For lipid droplet enumeration in the presence of C75, 5.0×10^4^ BHK-21 cells were seeded per well in 24-well plates containing a 1 cm^2^ coverslip and allowed to attach overnight. Cells were mock-infected or DENV-infected (MOI of 10). The inoculum was removed 1 h post-infection and 0.5 ml of fresh medium supplemented with 2% fetal bovine serum was added in the presence of 0, 5, 10, or 20 µM of C75. At the indicated time points post-infection, the slides were fixed and directly used for lipid droplet enumeration. Cell viability in the presence of C75 was determined by MTS assay (Cell titer 96®Aqueous Non-Radioactive Cell proliferation Assay, Promega). To evaluate the effect of C75 on DENV replication, the above protocol was used and the supernatants harvested at 24 and 48 h post-infection were used for virus quantification by plaque assay. For studies using the reporter virus carrying luciferase, a viral stock of mDV-R was first prepared by RNA transfection of BHK cells. This stock was used to infect cells in the presence of 0, 10, or 20 µM of C75. Luciferase activity was evaluated at 10, 24 and 48 h post-infection. After 48 h of infection, the supernatant was collected and used to evaluate the release of mDV-R particles by infecting fresh BHK cells in the absence of C75. Luciferase activity was then measured 48 h after infection.
